# Inflammatory immune mechanisms underlying the comorbidity of psoriasis and atherosclerosis: clinical implications

**DOI:** 10.3389/fimmu.2026.1902354

**Published:** 2026-09-09

**Authors:** Wen Xin, Yuanmeng Tian, Sha Wang, Shuo Zhang, Zhenlin Chen

**Affiliations:** Shaanxi University of Chinese Medicine, Xianyang, China

**Keywords:** atherosclerosis, endothelial dysfunction, IL-23/Th17 axis, psoriasis, systemic inflammation

## Abstract

**Background:**

Psoriasis (PSO) is a chronic immune-mediated inflammatory disease and an independent risk factor for cardiovascular disease (CVD), particularly atherosclerosis (AS). Both disorders share common immunopathological pathways characterized by persistent systemic inflammation and dysregulated interactions between innate and adaptive immunity.

**Methods:**

In this review, we summarize the shared immunoinflammatory mechanisms linking psoriasis and atherosclerosis and highlight potential therapeutic strategies targeting cutaneous manifestations and systemic comorbidities.

**Results:**

Dendritic cells (DCs), macrophages, neutrophils, and T lymphocytes contribute to a proinflammatory milieu that affects not only the skin but also the vasculature. The interleukin-23 (IL-23)/T helper 17 (Th17) axis plays a central role in inducing proinflammatory cytokines such as interleukin-17 (IL-17), tumor necrosis factor-alpha (TNF-α), and interleukin-1 beta (IL-1β), thereby promoting endothelial dysfunction, oxidative stress, and vascular inflammation. In turn, activation of pattern-recognition receptors and the NOD-like receptor pyrin domain-containing 3 (NLRP3) inflammasome amplifies inflammatory responses, linking keratinocyte (KC) hyperproliferation to vascular injury. Collectively, these processes may form a self-perpetuating inflammatory network that links cutaneous inflammation to CVD.

**Conclusion:**

In this review, we provide a comprehensive overview of the immunological interactions underlying the psoriasis-atherosclerosis connection and highlight potential therapeutic strategies targeting both cutaneous manifestations and systemic comorbidities.

## 1 Introduction

Psoriasis (PSO) is a chronic, immune-mediated, inflammatory, systemic skin disease induced by interactions between genetic susceptibility and environmental factors and typically manifests as scaly erythema or papules (1). In recent years, increasing evidence has shown that PSO is essentially an immune-mediated systemic inflammatory disease. In 2021 alone, approximately 5.1 million new cases were diagnosed worldwide, representing a substantial global disease burden (2). Patients with PSO commonly present with multisystem comorbidities, including mental health disorders (3), musculoskeletal disorders (4), and metabolic diseases such as cardiovascular disease (CVD), metabolic syndrome, and diabetes mellitus (5). These comorbid features have shifted the understanding of PSO from a skin-limited disorder to a systemic inflammatory disease. In this context, substantial epidemiological evidence has established PSO as an independent risk factor for CVD (6). CVD is primarily driven by atherosclerosis (AS) (7). Multiple large-scale cohort studies and meta-analyses have shown that patients with PSO have a significantly increased risk of coronary artery disease, hypertension, angina pectoris, and hypercholesterolemia (8).

These findings suggest that PSO and AS may not be merely a statistical coincidence but instead may share a common pathophysiological basis driven by systemic immune activation. Therefore, elucidating the shared immunoinflammatory mechanisms underlying these two disorders is of great clinical importance.

## 2 Innate immunity: initiation of tissue injury and amplification of inflammation

### 2.1 Convergent activation of sentinel cells: shared features of dendritic cells across different tissues

Dendritic cells (DCs) can be divided into plasmacytoid DCs (pDCs), conventional DC type 1 (cDC1s), and conventional DC type 2 (cDC2s) (9). These cells serve as major inflammatory initiation hubs in both diseases. In the early stage of PSO, pDCs initiate inflammatory responses by activating the Toll-like receptor (TLR)/nuclear factor-kappa B (NF-κB) signaling pathway and producing type I interferons (IFN-α/β) (10). Conventional DCs (cDCs), in turn, are the major source of interleukin-23 (IL-23) and are responsible for T helper 17 (Th17)-cell polarization, thereby driving disease progression (11). In parallel, myeloid dendritic cells (mDCs), together with other DC subsets triggered by neuropeptides and calcitonin gene-related peptide (CGRP), further enhance T helper 1/17/22 (Th1/Th17/Th22) responses through the release of interleukin-12 (IL-12) and IL-23, thereby sustaining and aggravating psoriatic inflammation (12).

A functionally similar cellular network also exists in AS (13). During atherogenesis, conventional dendritic cells (cDC1s and cDC2s) are key antigen-presenting cells that activate naive T cells. In the aortic wall, cDCs can stimulate CD4+ T cells to release interferon-gamma (IFN-γ) and tumor necrosis factor-alpha (TNF-α), thereby aggravating chronic inflammation and promoting foam cell formation (13). pDCs further accelerate lesion progression by producing type I interferons (IFN-I) and presenting antigens through major histocompatibility complex class II (MHC-II), thereby enhancing CD4+ T cell responses (13, 14). Thus, DCs in the skin and vasculature share a common pathogenic pattern, linking danger signal recognition to T cell instruction, and serve as initiating hubs in the psoriasis-atherosclerosis comorbidity network.

### 2.2 The double-edged role of effector amplifiers: pathogenic division of labor between neutrophils and macrophages

Neutrophils and macrophages together constitute a shared effector layer that amplifies inflammation and drives tissue injury in both PSO and AS. In cutaneous and vascular lesions, these cells do not merely perform overlapping effector functions; instead, they jointly sustain chronic inflammatory self-amplification through neutrophil extracellular traps (NETs), pyroptosis, interleukin-1 beta (IL-1β)/interleukin-18 (IL-18) release, and trained immunity-like remodeling in monocytes and macrophages. Neutrophils represent a functionally heterogeneous immune cell population capable of either suppressing or enhancing immune responses (15). In PSO, neutrophils drive disease progression through multiple mechanisms, including the release of reactive oxygen species (ROS), NETs, and interleukin-17A (IL-17A), thereby exacerbating tissue injury (16–18). A central mechanism involves a positive feedback amplification loop: neutrophil-derived S100 calcium-binding protein A9 (S100A9) triggers DCs to produce interleukin-23 (IL-23), thereby activating the IL-23/interleukin-17 (IL-17) axis; this axis, in turn, induces keratinocytes (KCs) to upregulate S100A9 expression and further promotes neutrophil recruitment (19). Moreover, neutrophil-derived gasdermin D (GSDMD) can mediate pyroptosis of neutrophils and surrounding cells, leading to the release of IL-1β and IL-18 and the further recruitment of inflammatory cells (20, 21). Neutrophils contribute significantly to plaque instability during AS, including plaque rupture and erosion, by releasing ROS and proteolytic enzymes that damage the fibrous cap (22). Oxidized low-density lipoprotein (ox-LDL) promotes neutrophil extracellular trap (NET) formation through the TLR-protein kinase C (PKC) signaling pathway (23), and these NETs serve as autoantigens that activate DCs to release IFN-α (24). Similar to GSDMD-mediated pyroptosis in psoriatic lesions, NETs activate the absent in melanoma 2 (AIM2) inflammasome in macrophages, leading to the production of IL-1β and IL-18 and thereby promoting unstable atherosclerotic lesions (25). This process forms a positive feedback loop involving pyroptosis, NET formation, and IL-1β, linking cutaneous and vascular inflammation. Thus, neutrophils are not only terminal effector cells but also important amplifiers that perpetuate chronic inflammation in both diseases.

Macrophages exhibit substantial plasticity in PSO and AS. M1 polarization is a crucial factor in the pathogenesis of PSO. Polarized M1 macrophages exacerbate KC hyperproliferation and neutrophil infiltration through the secretion of proinflammatory cytokines, such as TNF-α and IL-23 (26). By contrast, M2 macrophages exert anti-inflammatory effects. Their polarization may be facilitated by interleukin-35 (IL-35) released from regulatory T (Treg) cells and regulatory B (Breg) cells (27) and sustained through signal transducer and activator of transcription 6 (STAT6)-dependent signaling (27, 28). In AS, macrophages take up oxidized low-density lipoprotein (oxLDL) through scavenger receptors and subsequently transform into foam cells. M1 macrophages secrete large amounts of proinflammatory cytokines, ROS, and matrix metalloproteinases (MMPs), thereby accelerating plaque formation and destabilization (29). In contrast, M2 macrophages, including M2a, M2b, M2c, and Mhem subsets, exert anti-atherosclerotic effects mainly through the secretion of interleukin-10 (IL-10) and other anti-inflammatory mediators. M4, Mox, and M2d macrophages, which are polarized through the interleukin-6 (IL-6)/TLR-adenosine A2A receptor (A2AR) signaling pathway, promote plaque angiogenesis and growth by producing vascular endothelial growth factor (VEGF) (29, 30). Recent studies have shown that enhanced macrophage autophagy, such as increased autophagy-related 14 (ATG14) expression, can suppress inflammation and increase Treg abundance, thereby alleviating AS (31). Notably, upstream signals that promote M1 polarization, including IFN-γ, IL-17A, oxidized lipids, and microbial components, coexist in both diseases. This suggests that the same systemic inflammatory milieu may simultaneously bias macrophages in the skin and vasculature toward a proinflammatory phenotype, thereby providing a cellular basis for inter-organ inflammatory amplification.

### 2.3 Integration of danger signals: pathogenic roles of the NLRP3 inflammasome and pattern recognition receptors

The core of innate immunity comprises multiple pattern recognition receptors (PRRs), including NOD-like receptors (NLRs; such as NLRP1 and NLRP3), AIM2, TLRs, and pyrin inflammasomes (32). These PRRs and inflammasomes function as molecular hubs that convert cellular damage signals into systemic cytokine-driven inflammatory responses. In PSO, downregulation of DNase1L2 impairs DNA clearance in lesional skin, leading to persistent activation of the cyclic GMP-AMP synthase (cGAS)-stimulator of interferon genes (STING) axis and the AIM2 inflammasome. This process induces multiple forms of programmed cell death, including pyroptosis, thereby releasing additional DNA and establishing a self-amplifying inflammatory cycle (33). Meanwhile, danger-associated signals derived from injured KCs, such as ATP, ROS, and cathelicidin antimicrobial peptide LL-37 (LL-37), activate the NOD-like receptor pyrin domain-containing 3 (NLRP3) inflammasome, promoting the maturation of IL-1β and IL-18 and further enhancing Th17-mediated immune responses (34). In AS, cholesterol crystals, saturated fatty acid accumulation, and K+ efflux act as stimuli that induce NLRP3 inflammasome assembly and activation (35). This process culminates in caspase-1 activation, promotes the maturation and secretion of IL-1β and IL-18, and drives GSDMD cleavage, ultimately inducing pyroptotic cell death (36). Beyond inflammatory cytokine production, NLRP3 activation in monocytes can also induce phenotypic switching of vascular smooth muscle cells (VSMCs), thereby affecting plaque stability (37).

### 2.4 Neuroimmune integrators: mast cells and innate lymphoid cells

Mast cells (MCs) and innate lymphoid cells (ILCs) are located at the crossroads of the neurological, psychiatric, and immunological systems, making their role in linking PSO and AS particularly important. In PSO, neuropeptides activate MCs via the Mas-related G protein-coupled receptor X2 (MRGPRX2), resulting in the production of TNF, VEGF, and multiple chemokines (38). Activated MCs also produce chemokine (C-X-C motif) ligand 1 (CXCL1) and interleukin-8 (IL-8), facilitating neutrophil recruitment and Munro microabscess formation (39). Beyond innate immune activation, MCs modulate adaptive immunity through antigen presentation and the OX40/OX40 ligand (OX40L) axis, thereby promoting Th17 and Th22 polarization (38). Importantly, MCs form a self-reinforcing neuroimmune circuit with peripheral nerve fibers: neuropeptides drive MC activation through receptors such as MRGPRX2, whereas MC-derived pruritogenic mediators reciprocally sensitize neighboring neurons (40). In addition, norepinephrine can also promote MCs toward a proinflammatory phenotype, thereby further enhancing inflammatory responses (38). In AS, MCs infiltrate atherosclerotic plaques and secrete tryptase and chymase. These proteases degrade the extracellular matrix (ECM) and convert pro-matrix metalloproteinases (pro-MMPs) into their active forms (41). MC-derived mediators also induce SMC apoptosis, inhibit smooth muscle cell (SMC) proliferation, enhance high-density lipoprotein (HDL) degradation, impair cholesterol efflux from foam cells, and stimulate macrophage apoptosis, thereby expanding the necrotic core (42). MCs are involved in a self-amplifying neuroimmune positive-feedback loop that not only initiates cutaneous inflammation but also exacerbates vascular lesions, suggesting a key biological basis for skin-vascular neuroimmune coupling.

Tissue-resident ILCs are classically divided into ILC1s, ILC2s, and ILC3s based on their unique transcription factors and cytokine signatures (43). ILC3s are early drivers of disease in PSO. They also directly affect KCs by triggering their proliferation and initiating a self-amplifying inflammatory feed-forward loop through the production of IL-17A and interleukin-22 (IL-22) (44). Moreover, IL-23/signal transducer and activator of transcription 3 (STAT3) signaling participates in IL-17 production by ILC3s, which is markedly elevated and plays an essential role in psoriatic inflammation (45). Different ILC subsets have distinct functions in AS. ILC1s enhance plaque development through T-box transcription factor expressed in T cells (T-bet)-dependent production of IFN-γ (46). In contrast, ILC2s usually antagonize type 1 immune responses through GATA3-mediated production of type 2 cytokines, including interleukin-4 (IL-4), interleukin-5 (IL-5), IL-10, and interleukin-13 (IL-13), thereby exerting protective effects (46). Interleukin-33 (IL-33) generated from ILC2s can also promote T helper 2 (Th2)-associated cytokines and ox-LDL antibodies, improve fatty acid metabolism in adipose tissue and ultimately decrease the progression of AS, as shown in animal studies (47).

Thus, MCs and ILCs do not simply carry out the same inflammatory programs in PSO and AS; rather, they are reprogrammed within distinct tissue niches. In the skin, the neuropeptide-MRGPRX2-IL-23/IL-17/IL-22 network skews them toward neurogenic and epidermal inflammatory amplification, whereas signals from ox-LDL, cholesterol crystals, hypoxia, and plaque remodeling in the vascular wall drive them toward lipid-associated inflammation, ECM degradation, and plaque destabilization. This “neuroimmune-metabolic niche switching” may represent a key mechanism underlying the shared involvement of these immune cells in PSO and AS despite their divergent pathological outcomes. The pathogenic roles of these innate immune cells (**Table 1**) in PSO and AS are mainly reflected in acute or subacute inflammatory amplification. However, the chronic and relapsing nature of both diseases suggests a deeper layer of immune memory, namely trained immunity, which allows inflammatory responses to persist beyond the initial stimulus and to be rapidly recalled upon secondary challenge.

**Table 1:** Parallel pathogenic features and shared immunoinflammatory circuits in psoriasis and atherosclerosis.

Pathogenic nodes	Key mediators	Manifestations in psoriasis	Manifestations in cardiovascular diseases	References
Initiating sentinel cells (dendritic cells)	IL-23, IL-12, IFN-α	Activation of pDCs and cDCs, polarizing Th17, Th1 and Th22 cells to initiate psoriatic inflammation	cDCs induce Th1/Th17 responses and can transform into foam cells	(11, 13)
Tissue damage executive layer (neutrophils)	ROS, NETs, IL-17A, S100A9, IL-1β	Neutrophils release ROS and NETs to damage tissues; the S100A9-IL-23-IL-17 axis forms a positive feedback loop to amplify inflammation	Neutrophils release ROS and proteases to disrupt plaques; ox-LDL induces NETs to activate inflammasomes and amplify vascular inflammation.	(16–19, 22, 23)
Inflammatory amplifier (macrophages)	TNF-α, IL-23, IL-10	Macrophages polarize into M1 phenotype and secrete pro-inflammatory cytokines to aggravate skin lesions, while M2 macrophages exert anti-inflammatory effects	Macrophages phagocytose ox-LDL to form foam cells; M1 macrophages promote plaque progression, whereas M2 macrophages stabilize plaques.	(26–30)
Systemic inflammation converter (NLRP3/AIM2 inflammasome)	IL-1β, IL-18, ATP, ROS, Cholesterol crystals	Activated by damage signals, it cleaves IL-1β and IL-18 to potentiate Th17 responses	Activated by cholesterol crystals, it mediates pyroptosis and affects plaque stability	(34, 36)
Neuro-immune coupling (mast cells)	TNF, VEGF, Tryptase, IL-8	Neuropeptides trigger the release of inflammatory mediators and regulate Th17/Th22 cell differentiation.	Mast cells secrete proteases within plaques to degrade ECM and exacerbate plaque instability	(38, 39, 41, 42)
Local inflammation initiation and regulation (innate lymphoid cells)	IL-17A, IL-22, IFN-γ, IL-33	ILC3s secrete IL-17A and IL-22 to initiate the cutaneous inflammatory cycle.	ILC1s promote plaque formation, while ILC2s exert protective effects.	(44–46)
Skin-vascular inflammatory coupling (Th17 cells)	IL-17A, IL-17F, IL-22, IL-23	IL-23 drives cell expansion and induces keratinocyte proliferation via IL-17A secretion.	Secretion of IL-17A damages endothelium and exacerbates vascular inflammation and plaque instability.	(48, 49)
Inflammatory amplification arm of the IL-23/Th17 axis (Th1 cells)	IFN-γ, TNF-α, IL-12	Secrete IFN-γ and TNF-α to enhance Th17 polarization and amplify cutaneous inflammation	Secrete IFN-γ and TNF-α to activate macrophages and impair plaque stability	(50, 51)
Tissue remodeling and regulation (Th22 cells)	IL-22, STAT3	Secrete IL-22 to drive epidermal thickening and skin barrier dysfunction	Secrete IL-22 to activate STAT3, promote Th17 expansion, and SMC dedifferentiation	(52–54)
Immune imbalance (B lymphocytes)	IL-10, IgM, IgG、ox-LDL	Reduced IL-10 secretion by Bregs leads to Th17/Treg imbalance	B1 and Breg cells stabilize plaques, whereas B2 cells exert proinflammatory effects and aggravate atherosclerosis	(55–57)
Immune imbalance (regulatory T cells)	FOXP3, IL-10, TGF-β	Decreased FOXP3 stability impairs immunosuppressive function, resulting in Th17/Treg imbalance.	Inhibits foam cell formation, promotes M2 polarization, suppresses Th1/Th17 responses and stabilizes plaques	(58–60)
Linkage of cutaneous vascular inflammation (Th9 cells)	IL-9, IL-9R, STAT3	Secrete IL-9 to promote angiogenesis and amplify Th17-mediated inflammation	IL-9/STAT3 signaling impairs endothelium and accelerates plaque progression	(52, 61)

PSO, psoriasis; CVD, cardiovascular disease; AS, atherosclerosis; IL, interleukin; TNF, tumor necrosis factor; IFN, interferon; ROS, reactive oxygen species; NETs, neutrophil extracellular traps.

### 2.5 Trained immunity: persistent inflammatory memory in the comorbidity of psoriasis and atherosclerosis

#### 2.5.1 Concept and classification of trained immunity: dual-layer memory in central and peripheral compartments

**Figure 1:**
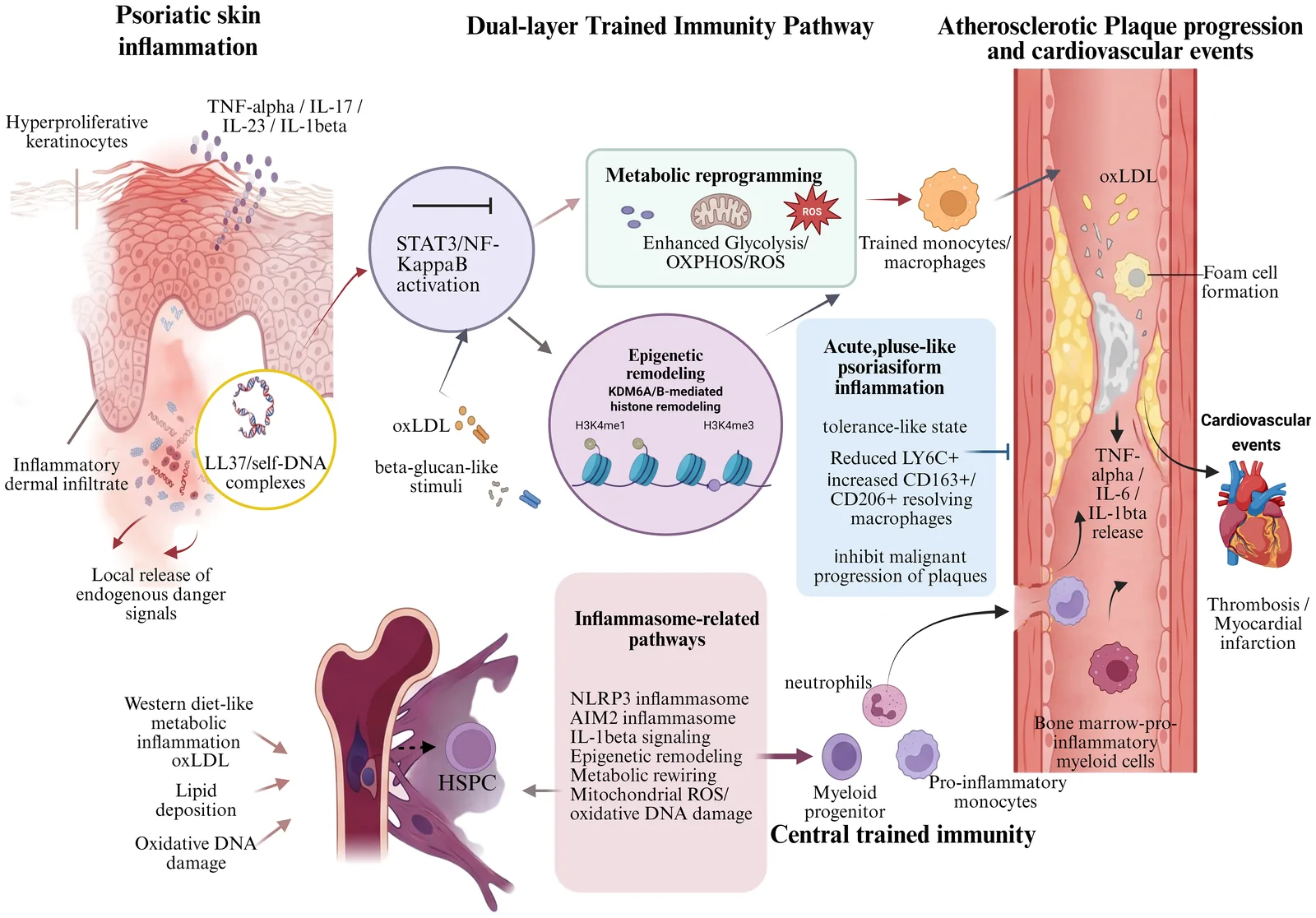
Mechanisms of trained immunity in psoriasis-associated atherosclerosis: a dual peripheral-central pathway linking cutaneous inflammation to cardiovascular events. LL37/self-DNA complexes in psoriatic lesions (peripheral pathway) and oxLDL in the vascular wall (central pathway) can each induce trained immunity in monocytes/macrophages and HSPCs, respectively. This process involves KDM6A/B-mediated H3K27me3 demethylation, enrichment of H3K4me1/3, enhanced glycolysis, and increased mitochondrial ROS production, thereby maintaining pro-inflammatory genes in an open, transcriptionally poised state. Upon secondary stimulation, trained myeloid cells release TNF-α, IL-1β, and IL-6, accelerating foam cell formation, inflammatory infiltration, and plaque progression, ultimately increasing the risk of cardiovascular events. Notably, acute, IFN-I-dominant inflammation may induce a tolerance-like state in HSPCs and increase the proportion of CD163^+^/CD206^+^ reparative macrophages, thereby limiting plaque aggravation. LL37, human cathelicidin antimicrobial peptide LL37; DNA, deoxyribonucleic acid; oxLDL, oxidized low-density lipoprotein; HSPCs, hematopoietic stem and progenitor cells; KDM6A/B, lysine-specific demethylase 6A/6B; H3K27me3, histone H3 lysine 27 trimethylation; H3K4me1/3, histone H3 lysine 4 monomethylation/trimethylation; ROS, reactive oxygen species; TNF-α, tumor necrosis factor-α; IL-1β, interleukin-1β; IL-6, interleukin-6; IFN-I, type I interferon; CD163, cluster of differentiation 163; CD206, cluster of differentiation 206/mannose receptor. Created with BioRender.com.

Trained immunity is a memory-like functional state of the innate immune system. Its defining feature is that innate immune cells and their progenitors acquire enhanced and more durable responses to secondary stimulation after an initial challenge through long-term epigenetic reprogramming and metabolic rewiring (62). According to the site of memory establishment and maintenance, trained immunity can be divided into central trained immunity and peripheral trained immunity. Central trained immunity occurs primarily in bone marrow hematopoietic stem and progenitor cells (HSPCs), in which inflammatory or microbial signals can directly reprogram HSPCs and transmit this “memory” to downstream myeloid cells derived from them, thereby generating systemic and sustained immune potentiation. In contrast, peripheral trained immunity mainly takes place in tissue-resident immune cells, such as macrophages and microglia, which can independently acquire a persistent proinflammatory phenotype in response to local microenvironmental stimuli without relying on bone marrow progenitors (63). This dual-layer memory model (**Figure 1**) provides a new perspective for understanding how chronic, relapsing inflammation in PSO may spill over into systemic vascular diseases such as AS. It suggests that local inflammatory signals in the skin may shape a long-lasting and readily triggerable cardiovascular inflammatory background through both peripheral and central pathways.

#### 2.5.2 Peripheral trained immunity: metabolic reprogramming and pro-inflammatory memory in monocytes/macrophages

In PSO, LL-37 (human cathelicidin antimicrobial peptide)/self-DNA complexes can be taken up by monocytes as endogenous danger signals within lesional skin and activate the STAT3 and NF-κB pathways, leading to enhanced glycolysis, oxidative phosphorylation, and ROS generation. In parallel, these complexes can upregulate the activity of lysine-specific demethylase 6A/6B (KDM6A/B), thereby selectively removing the repressive histone mark histone H3 lysine 27 trimethylation (H3K27me3) from the promoter regions of proinflammatory genes such as IL-6 and TNF-α, while increasing the enrichment of the activating mark H3K4me3. This epigenetic remodeling maintains these genes in a more accessible and readily inducible state, conferring a persistent trained immunity phenotype on monocytes. These findings suggest that trained immunity in PSO is not confined to local epidermal cells or the lesional microenvironment but may extend through peripheral monocytes as a form of systemic inflammatory memory, providing a potential mechanism by which cutaneous inflammation spills over into vascular inflammation (64). Similarly, in AS, oxLDL serves as a key stimulus that activates hypoxia-inducible factor 1-alpha (HIF-1α) via the NF-κB/protein kinase B (Akt)/mechanistic target of rapamycin (mTOR) pathway, thereby inducing metabolic reprogramming in monocytes/macrophages centered on enhanced glycolysis. This process is also accompanied by the sustained retention of activating histone marks, such as histone H3 lysine 4 monomethylation (H3K4me1) and H3K4me3 (65). As a result, these cells can mount a stronger inflammatory response upon secondary stimulation at a lower activation threshold.

A key advance is that the functional state of macrophages under trained immunity cannot be fully captured by the classical M1 (pro-inflammatory)/M2 (anti-inflammatory) polarization framework. Studies have shown that oxLDL- or β-glucan-induced trained immunity does not always lead to a selective increase in M1 markers; in some cases, M2 markers are also upregulated, or no significant differences are observed between the two phenotypes (66). These seemingly contradictory findings suggest that trained immunity does not simply drive monocytes/macrophages toward an M1- or M2-polarized state but instead establishes a more upstream reactive poised state. In this state, cells acquire a lower activation threshold, greater transcriptional plasticity, and enhanced secondary responsiveness through metabolic rewiring and chromatin opening. Whether the eventual outcome is a proinflammatory, lipid-uptake, tissue-reparative, or mixed phenotype depends on the specific microenvironment and the nature of the secondary stimulus encountered by the cells. In other words, trained immunity provides a permissive, readily activatable substrate, whereas M1/M2-like phenotypes represent distinct outputs generated from that substrate in different lesion settings.

Based on these observations, we propose that the exacerbation of atherosclerosis by systemic inflammation associated with psoriasis may arise not only from an increased proportion of M1 macrophages, but also from the preconditioning of circulating monocytes by signals such as LL-37/self-DNA. Once recruited to the vascular wall and exposed to secondary insults such as oxLDL and cholesterol crystals, these cells, now in a primed state, amplify local inflammation more strongly, thereby accelerating foam cell formation and plaque progression. This may represent a key peripheral immune memory pathway through which PSO spills over into vascular inflammation.

#### 2.5.3 Central trained immunity: reprogramming of bone marrow hematopoietic stem and progenitor cells and sustained myeloid output

Beyond peripheral monocytes, psoriasis-associated inflammatory signals may also reach the bone marrow and reshape myeloid output at its source by inducing central trained immunity. In clonal hematopoiesis driven by the *JAK2^V617F^* (*JAK2^VF^*) mutation, activation of the AIM2 inflammasome has been identified as an important mechanism that exacerbates AS. *JAK2^VF^* mutations can increase mitochondrial ROS production and oxidative DNA damage in macrophages, thereby activating the AIM2 inflammasome (25). Notably, inflammasomes are not only amplifiers of acute inflammation, but also key executors in the establishment and maintenance of trained immunity. Animal studies have shown that psoriasis-like stimuli can confer inflammatory memory on cutaneous epithelial stem cells, a process that strictly depends on AIM2 inflammasome signaling and IL-1β signaling and is accompanied by hypomethylation of the AIM2 promoter region (67).

In AS, a similar “damage recognition-inflammasome activation-trained immunity maintenance” axis also operates (66).

Therefore, the NLRP3 and AIM2 inflammasomes may constitute a shared “damage-inflammation-memory” converter in PSO and AS. They can integrate upstream stimuli from distinct tissue sources, including KC injury, LL-37/self-DNA complexes, vascular lipid deposition, and oxidative DNA damage, and convert them into an IL-1β-driven inflammatory cascade. Through metabolic remodeling and epigenetic reprogramming, they further sustain a chronic, relapsing, and systemic inflammatory state.

It should be emphasized that the impact of psoriatic inflammation on vascular lesions is not invariably proinflammatory or proatherogenic. Some animal studies have shown that an acute and self-limited psoriasis-like inflammatory flare can induce a tolerance-like state in HSPCs through IFN-I signaling. As a result, the influx of Ly6C+ inflammatory monocytes/macrophages into the vascular wall is reduced, whereas the proportion of cluster of differentiation 163 (CD163+)/cluster of differentiation 206 (CD206+) pro-resolving or reparative macrophages increases, thereby suppressing adverse plaque progression under specific conditions (68). This does not contradict clinical observations that chronic PSO increases cardiovascular risk. The key distinction may lie in the pattern of inflammatory exposure: acute, pulse-like, IFN-I-dominant inflammation is more likely to induce compensatory central tolerance, whereas chronic and recurrent inflammation driven by IL-23/IL-17/TNF-α/IL-1β signaling, together with metabolic inflammation, may be more likely to cross a “tolerance-to-training” transition threshold, thereby promoting myeloid-biased trained immunity and sustained vascular inflammation. Therefore, whether PSO promotes AS by inducing central trained immunity, and under what conditions, remains to be confirmed by more precise longitudinal studies.

## 3 Adaptive immune mechanisms in psoriasis and atherosclerosis

### 3.1 IL-23/Th17 signaling: the core immunologic nexus linking skin and vascular inflammation

**Figure 2:**
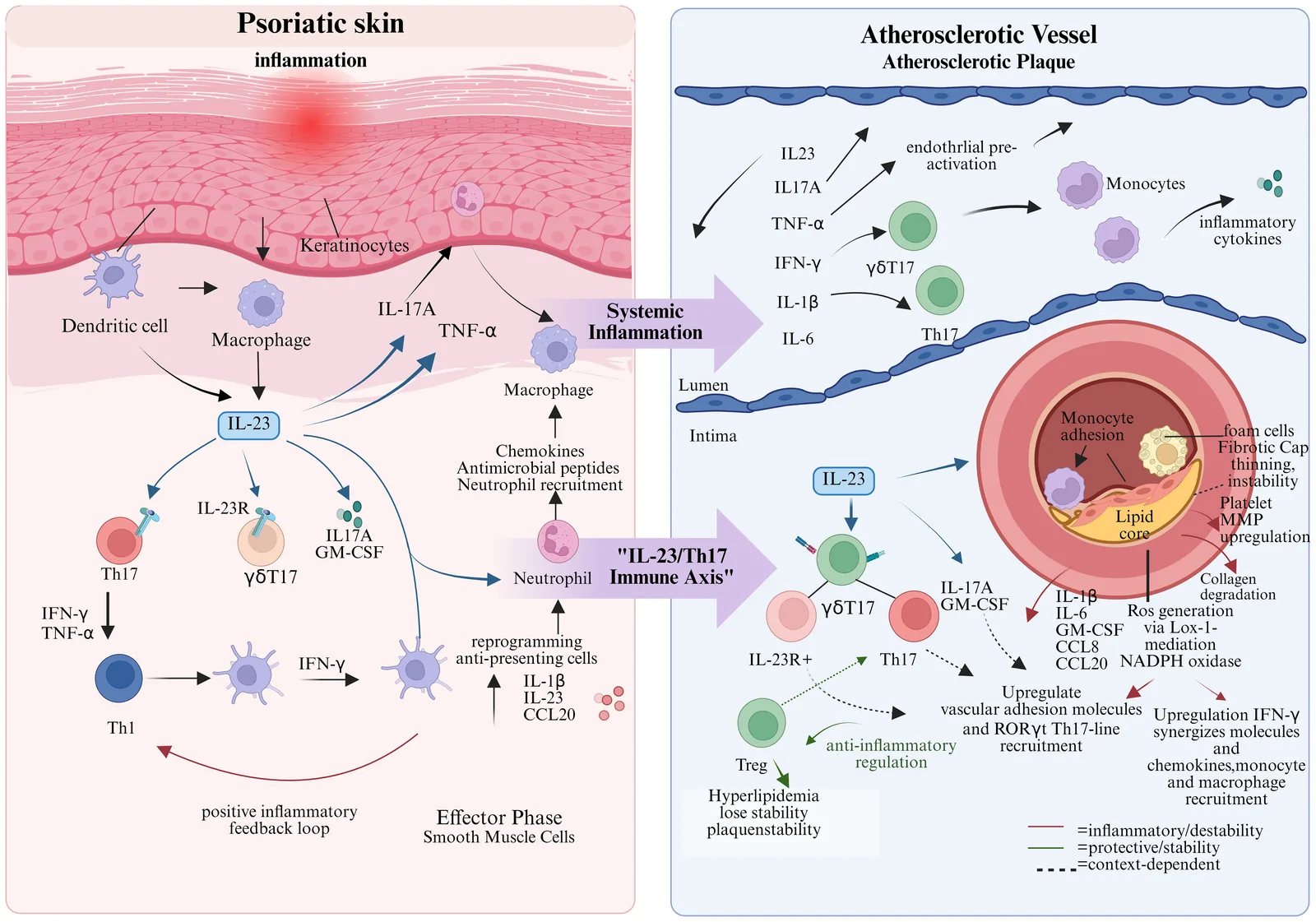
Core driving axis: parallel pathogenic mechanisms of the IL-23/Th17 pathway in psoriatic skin inflammation and atherosclerotic vascular injury. In psoriatic skin, interactions among KCs, DCs, and macrophages promote the release of IL-23 and activate Th17 and γδT17 cells, thereby inducing the production of inflammatory mediators such as IL-17A, TNF-α, and IFN-γ. This process establishes a positive inflammatory feedback loop that further promotes KC activation, immune cell recruitment, and the persistence of cutaneous inflammation. These inflammatory mediators may enter the circulation and contribute to the maintenance of systemic inflammation. In the vascular system, IL-23/Th17-related inflammatory signals can induce endothelial cell preactivation and promote monocyte adhesion, migration, and differentiation into macrophages. Following lipid uptake, macrophages become foam cells and contribute to lipid core expansion and atherosclerotic plaque formation. Meanwhile, inflammatory mediators, including IL-17A, granulocyte-macrophage GM-CSF, IL-1β, IL-6, and CCL20, can further amplify vascular inflammation by promoting ROS generation, NADPH oxidase activation, and collagen degradation, leading to fibrous cap thinning and reduced plaque stability. Thus, the IL-23/Th17 immune axis may represent an important immunological bridge linking systemic inflammation in PSO with the progression of AS. IL-23, interleukin-23; Th17, T helper 17; γδT17, interleukin-17-producing gamma delta T cells; IL-17A, interleukin-17A; TNF-α, tumor necrosis factor-alpha; IFN-γ, interferon-gamma; GM-CSF, granulocyte-macrophage colony-stimulating factor; IL-1β, interleukin-1 beta; IL-6, interleukin-6; CCL20, C-C motif chemokine ligand 20; ROS, reactive oxygen species; NADPH, nicotinamide adenine dinucleotide phosphate. Created with BioRender.com.

The IL-23/Th17 axis not only plays a vital role in the pathogenesis of PSO but also serves as an important link between cutaneous and vascular inflammatory responses (**Figure 2**). IL-23 is highly expressed in psoriatic lesions, and its expression levels directly correlate with disease severity (69). In addition, IL-23 can act directly on macrophages and trigger the production of proinflammatory mediators such as IL-17A (48), which exacerbate inflammatory responses. The role of IL-23 in AS seems to depend on the setting. Exogenous IL-23 can stimulate interleukin-23 receptor-positive gamma delta T (IL-23R+ γδT) cells to secrete IL-17 and granulocyte-macrophage colony-stimulating factor (GM-CSF), which accelerates plaque formation (70). These seemingly contradictory findings suggest that the role of IL-23 in AS is not determined solely by its expression level but is instead jointly regulated by its site of action, target cell types, disease stage, and the specific mode of experimental intervention. Accordingly, systemic IL-23 deficiency may attenuate local vascular pro-inflammatory signaling while also impairing gut barrier-protective functions, ultimately exacerbating AS in certain models or at specific stages of disease.

Th17 cells represent the principal effector subset downstream of IL-23 signaling. Their differentiation depends on the transforming growth factor-beta 1 (TGF-β1)/IL-6 axis and the retinoic acid-related orphan receptor gamma t (RORγt)/Janus kinase (JAK)-STAT3 pathway (71), whereas their expansion and functional stabilization are driven by IL-23 (72). In PSO, Th1-derived IFN-γ further augments Th17 polarization and fuels a self-amplifying inflammatory cycle by reprogramming antigen-presenting cells to upregulate the production of interleukin-1 (IL-1), IL-23, and C-C motif chemokine ligand 20 (CCL20) (52). In AS, Th17/Treg immune imbalance constitutes a key pathogenic mechanism: Th17 cells promote plaque progression through the IL-23/RORγt axis, whereas Treg cells preserve plaque stability through anti-inflammatory effects (73). However, Treg cells are not a static protective cell population. In the context of hyperlipidemia, oxidative stress, and the inflammatory plaque microenvironment, forkhead box P3-positive (Foxp3+) Treg cells may lose stability or undergo phenotypic plasticity, giving rise to T-box transcription factor expressed in T cells-positive (T-bet+) Th1-like Treg cells (74). Therefore, restoring the Th17/Treg balance should not be understood merely as suppressing Th17 expansion, but should also include maintaining Treg stability and blocking pathological transitions among Th1, Th17, and Treg cells (71).

IL-17A, the effector cytokine in PSO, causes KCs to generate chemokines and antimicrobial peptides, which promote neutrophil recruitment and synergistically enhance local inflammation in concert with TNF-α (75, 76). In AS, IL-17A acts on endothelial cells, VSMCs, and macrophages to induce the release of inflammatory cytokines and chemokines, including IL-1β, IL-6, granulocyte colony-stimulating factor (G-CSF), GM-CSF, C-C motif chemokine ligand 8 (CCL8), and CCL20. IL-17A can also promote ROS production via nicotinamide adenine dinucleotide phosphate (NAD(P)H) oxidase, thereby inducing oxidative stress and endothelial dysfunction. In addition, IL-17A may facilitate apoptosis of human umbilical vein endothelial cells (HUVECs) and VSMCs, upregulate MMPs and degrade collagen, and promote foam cell formation and platelet activation through lectin-like oxidized low-density lipoprotein receptor-1 (LOX-1), thereby increasing plaque vulnerability and thrombotic risk. However, IL-17A also exhibits a “paradoxical” effect in AS. Some studies have suggested that IL-17A can promote collagen production by VSMCs, increase fibrous cap thickness, or attenuate inflammatory spread by limiting vascular cell adhesion molecule-1 (VCAM-1) expression and excessive Th1 responses (49). We consider that this discrepancy does not reflect a true contradiction, but rather a functional switch of IL-17A across different disease stages and microenvironments. During the early or active inflammatory phase, IL-17A is predominantly proatherogenic and plaque destabilizing; whereas during the chronic reparative phase, low-intensity and locally restricted IL-17A signaling may primarily contribute to plaque stabilization. Moreover, differences in experimental design may further magnify the apparent divergence in IL-17A function. Mice deficient in IL-17A (*IL-17A−/−*) lack IL-17A from embryonic development and may therefore develop compensatory immune adaptations that confound its direct effects, whereas short-term antibody blockade in adult animals more faithfully reflects the immediate inflammatory actions of IL-17A but is less informative regarding its long-term roles in immune homeostasis and tissue repair. Different mouse models, such as apolipoprotein E-deficient (*Apoe−/−)* and low-density lipoprotein receptor-deficient (*Ldlr−/−*) mice, also differ in lipid metabolism, plaque composition, and basal inflammatory status; similarly, the use of a Western diet versus a high-fat diet, as well as differences in feeding duration, may shift the corresponding stage of AS pathology. At the same time, the cellular source of IL-17A, including Th17 cells, γδT cells, or ILCs, may also determine its functional bias. Importantly, most current clinical studies remain cross-sectional or observational and can only suggest an association between IL-17A and AS risk or vascular inflammation, rather than establishing a clear causal relationship. Therefore, the genuine role of IL-17A in AS should be interpreted by integrating disease stage, cellular source, experimental model, and longitudinal clinical evidence.

Based on the evidence above, this study proposes a hypothesis that the IL-23/Th17 axis mediates the comorbidity-associated link between PSO and AS. In PSO, sustained activation of the IL-23/Th17 axis establishes a systemic inflammatory milieu that leaves the vascular endothelium in a preactivated state. When local vascular conditions include hyperlipidemia, oxLDL, enrichment of IL-1β, TNF-α, and IFN-γ, together with Treg instability, the IL-23/IL-17A axis may exceed a proinflammatory threshold, thereby driving gamma delta T17 (γδT17) and Th17 cell expansion, macrophage inflammatory activation, monocyte infiltration, matrix degradation, and plaque destabilization. By contrast, when IL-23 signaling primarily sustains intestinal IL-22 responses, antimicrobial peptide expression, and microbiota homeostasis, or when IL-17A mainly contributes to collagen deposition by VSMCs during the chronic phase, this axis may instead exert barrier-protective or plaque-stabilizing effects. Therefore, the ultimate outcome of the IL-23/Th17 axis is determined not by a simple increase or decrease in a single cytokine, but by the tissue niche, disease stage, accompanying inflammatory cytokine profile, and mode of intervention.

Beyond Th17 cells, Th1 cells may serve as an “inflammatory amplification arm” of the IL-23/Th17 axis, further reinforcing this cross-system inflammatory network. In psoriasis, stimulation by IL-12 promotes Th1 polarization through signal transducer and activator of transcription 1 (STAT1)/T-bet signaling, leading to the secretion of IFN-γ and TNF-α. Among these mediators, IFN-γ induces the production of IL-1 and IL-23, thereby promoting Th17 expansion and establishing a positive feedback loop (50). In AS, IL-17A can synergize with the Th1 signature cytokine IFN-γ to upregulate the expression of vascular adhesion molecules and chemokines, accelerating the infiltration of monocytes and macrophages into atherosclerotic plaques and thereby driving the inflammatory cascade (51). Therefore, dynamic imbalance among Th1, Th17, and Treg may constitute a key immunological basis by which psoriatic skin inflammation spills over into vascular inflammation and increases AS risk.

In summary, the IL-23/Th17 axis is not a simple linear proinflammatory pathway, but rather a cross-system immune network jointly regulated by the skin, gut, and vascular microenvironments.

### 3.2 Dual roles and microenvironment dependency: TNF-α and IFN-γ

Although TNF-α and IFN-γ are conserved Th1-type cytokines in both diseases, their actions are highly context dependent across tissues. TNF-α is a major proinflammatory cytokine in PSO, and genetic polymorphisms are associated with disease risk (77). Mechanistically, TNF-α binds to tumor necrosis factor receptor 2 (TNFR2) to regulate IL-17 production by γδT cells, enhance DC secretion of IL-23, and promote Th17 differentiation (78). In cooperation with IL-17A, TNF-α induces Dectin-1 expression and activates the spleen tyrosine kinase (Syk)/NF-κB pathway, thereby exacerbating KC proliferation (79). Thus, TNF-α is an important therapeutic target in PSO. In AS, TNF-α induces endothelial senescence (80), causes VSMC phenotype switching, and promotes foam cell formation (74). At the molecular level, TNF-α signaling is mediated via the NADPH oxidase 1 (NOX1)-ROS and leucine-rich repeat-containing protein 8A (LRRC8A) pathways (81). Extracellular vesicles (EVs) from monocyte-platelet aggregates further promote TNF-α-mediated vascular damage and plaque instability (82). Therefore, TNF-α has become an important therapeutic target in AS (83).

IFN-γ is a critical regulatory factor in PSO and is positively associated with disease severity. The pathogenic effects of IFN-γ are controlled by a multilayered regulatory network. At the upstream level, whey acidic protein four-disulfide core domain protein 12 (WFDC12) enhances Th1 differentiation and induces IFN-γ release (84). At the downstream level, IFN-γ inhibits epiplakin 1 (EPPK1), thereby compromising the epidermal barrier (85), and downregulates miR-149 to increase KC sensitivity to tumor necrosis factor-like weak inducer of apoptosis (TWEAK) (86). Interestingly, epidermal growth factor (EGF) has been shown to strongly antagonize the IFN-γ-induced proinflammatory transcriptional program, suggesting a potential endogenous regulatory mechanism within this pathway (87). IFN-γ is also a key proinflammatory mediator that is substantially elevated in AS (88). At the cellular level, IFN-γ promotes vascular endothelial cell senescence (89), enhances monocyte infiltration and polarization toward proinflammatory M1 macrophages, and drives VSMCs toward proliferative or migratory phenotypes. Together, these biological activities lead to impaired lipid clearance, foam cell formation, and abnormal apoptosis, ultimately contributing to necrotic core formation in vulnerable atherosclerotic plaques (90). IFN-γ activates the AIM2 inflammasome through the JAK2/STAT1 pathway (91), induces liver X receptor-alpha (LXR-α) degradation to suppress ATP-binding cassette subfamily G member 1 (ABCG1)-dependent cholesterol efflux (92), and upregulates indoleamine 2,3-dioxygenase (IDO) to modify tryptophan metabolism (93). Importantly, IFN-γ may also exert potential protective regulatory effects, for example, by inducing programmed death-ligand 1 (PD-L1) expression on B cells and thereby suppressing T follicular helper (Tfh) cell responses; however, its net effect remains predominantly proatherogenic (94). Genetic and pharmacological investigations have demonstrated that IFN-γ targeting delays disease development (88).

### 3.3 Regulators of tissue remodeling: IL-22 and Th22 cells

Th22 cells are a subset of CD4+ T cells characterized by IL-22 production and are important for epithelial barrier homeostasis and tissue repair. In PSO, circulating Th22 cells are markedly increased and correlate positively with disease severity (52, 95). Th22-derived IL-22, as an important downstream effector arm of IL-23-driven inflammatory responses, mediates IL-23-induced acanthosis and immune cell infiltration and contributes to cutaneous tissue remodeling and sustained inflammation (52). In AS, IL-22 activates the IL-6/STAT3 pathway, promotes Th17 proliferation and SMC dedifferentiation, and accelerates lesion progression (53).

IL-22 is a member of the IL-10 family and the IL-20 subfamily of cytokines and is produced predominantly by Th22 cells and a subset of Th17 cells in PSO. IL-22 binds to receptor complexes on KCs to activate the STAT3 signaling pathway, resulting in aberrant proliferation of KCs, blockade of terminal differentiation, and promotion of epidermal hyperplasia and barrier failure (54). Clinical studies have shown that circulating IL-22 levels are significantly increased in patients with PSO, whereas its endogenous antagonist, interleukin-22 binding protein (IL-22BP), is relatively deficient, resulting in abnormal accumulation of free IL-22 and further amplification of its biological effects (96). IL-22 also regulates PSO through complex non-coding RNA networks. On the one hand, IL-22 causes downregulation of miR-124-3p and inhibition of growth factor receptor-bound protein 2 (GRB2) expression, thereby regulating KC proliferation and inflammatory responses (97). IL-22 may potentially induce miR-21-3p transcription through direct or indirect mechanisms involving the STAT3 and NF-κB signaling pathways (98). Competing endogenous RNA (ceRNA) mechanisms, on the other hand, finely regulate IL-22-mediated KC growth. For example, circular RNA 0061012 (circ_0061012) (99) and circ_0060531 (100) serve as sponges for miR-194-5p and miR-330-5p, respectively, thereby relieving their suppressive effect on the downstream effector protein GRB2-associated binding protein 1 (GAB1) and promoting KC proliferation. IL-22 is also implicated in vascular wall remodeling in AS. Most studies show that IL-22 stimulates Th17 proliferation via activation of the IL-6/STAT3 pathway and also induces dedifferentiation and phenotypic switching of VSMCs, thereby accelerating plaque formation (53). IL-22 is also involved in disease development through several pathways, including the regulation of angiogenesis, inflammatory responses, and lipid metabolism (101). However, the role of IL-22 in AS is still debated. Although most studies support a pro-atherogenic role, other studies have demonstrated that IL-22 deficiency in Ldlr−/− mice paradoxically increases the levels of pro-atherogenic metabolites including gut-derived trimethylamine N-oxide (TMAO) and lipopolysaccharide (LPS). These apparently contradictory results show that the exact function of IL-22 in AS may be extremely context-dependent and strongly influenced by variations in experimental design, including the animal models and dietary settings (102). Collectively, Th22 cells and their effector cytokine IL-22 are involved in abnormal epidermal proliferation and barrier remodeling in PSO and are involved in AS development by modulating vascular inflammation and phenotypic switching of VSMCs, providing an important molecular link between cutaneous and vascular tissue remodeling.

### 3.4 Disruption of immune homeostasis: B-cell and Treg dysfunction

A considerable increase in the proportion of B cells in peripheral blood and lesional skin has been reported in PSO, and B cell activation correlates positively with disease severity (103). Several autoantigen targets have been identified, such as heterogeneous nuclear ribonucleoprotein A1 (hnRNP-A1) and LL-37 (104, 105), indicating the involvement of autoreactive B cells in disease development. Bregs, on the other hand, exert core anti-inflammatory functions by secreting IL-10, promoting Treg proliferation, and blocking Th17 differentiation (55). Additional investigations have shown that Breg skin homing is dependent on α4β1 integrin (106). Clinical studies show that IL-10+ Bregs are markedly decreased in the peripheral blood of patients with PSO, and their levels are negatively associated with disease severity and the abundance of pathogenic T cells (107). Therapy with non-specific B cell depletion may further exacerbate disease development due to concomitant loss of protective Bregs, underscoring the necessity of sustaining Breg function in therapeutic interventions (103). B cells have a dual role in AS. Bregs can suppress inflammation and stabilize plaques (108). B1 cells and marginal zone B2 cells generate immunoglobulin M (IgM) antibodies against oxLDL that impede lipid uptake by macrophages and prevent foam cell development (56). Conversely, disease progression is driven by pathogenic subsets, mainly B2 cells, which produce proinflammatory antibodies such as immunoglobulin G (IgG) and immunoglobulin E (IgE). Fcγ receptors on macrophages bind IgG and trigger proinflammatory cytokine responses (57).

Tregs, defined by the CD4+ Foxp3+ phenotype, are immunosuppressive cells that are crucial for the maintenance of cutaneous immune homeostasis. Among these cells, peroxisome proliferator-activated receptor gamma-positive (PPARγ+) Tregs can regulate psoriatic inflammation by inhibiting IL-17A+ γδ T cells (109). In PSO, Tregs often exhibit impaired immunosuppressive function due to downregulation of the ecto-5’-nucleotidase (CD73)/adenosine monophosphate-activated protein kinase (AMPK) pathway, increased microRNA-210 (miR-210) expression, reduced FOXP3 stability, and aberrant activation of STAT3, thereby driving Th17/Treg imbalance and exacerbating inflammation (58). Therefore, restoration of Treg function and correction of Th17/Treg imbalance have become potential therapeutic strategies for PSO. In AS, Tregs exert anti-atherogenic effects through multiple mechanisms, including inhibition of macrophage lipid accumulation (59), promotion of M1-to-M2 macrophage polarization (60), direct or indirect inhibition of Th1 and Th17 immune responses (59), regulation of B-cell responses (59), and modulation of the maturation and function of antigen-presenting cells (59). Collectively, these mechanisms confer a protective role in atherosclerotic disease. Both PSO and AS are characterized by an imbalance between regulatory and pathogenic immune cell populations, resulting in an immunological milieu that supports persistent inflammation.

### 3.5 Th9 cells: a cutaneous-vascular inflammatory potential amplifier

T helper 9 (Th9) cells are a functional subset of cluster of differentiation 4-positive (CD4+) helper T cells. They primarily exert their effects by secreting interleukin-9 (IL-9) and, under inflammatory conditions, producing effector molecules such as TNF-α and granzyme B. In psoriasis, accumulating evidence suggests that IL-9 may promote angiogenesis, enhance inflammatory responses in KCs, and potentially amplify Th17-related responses, thereby contributing to cutaneous inflammation (52). Clinical studies have shown that peripheral blood Th17, Th22, Th9, and cytotoxic T cells are significantly elevated in patients with severe PSO (110). These findings support the notion that the Th9/IL-9 axis may participate in systemic immune activation in PSO; however, the current evidence is largely correlative and remains insufficient to establish this axis as an independent driver of disease onset or of skin-to-vascular inflammatory spillover.

**Figure 3:**
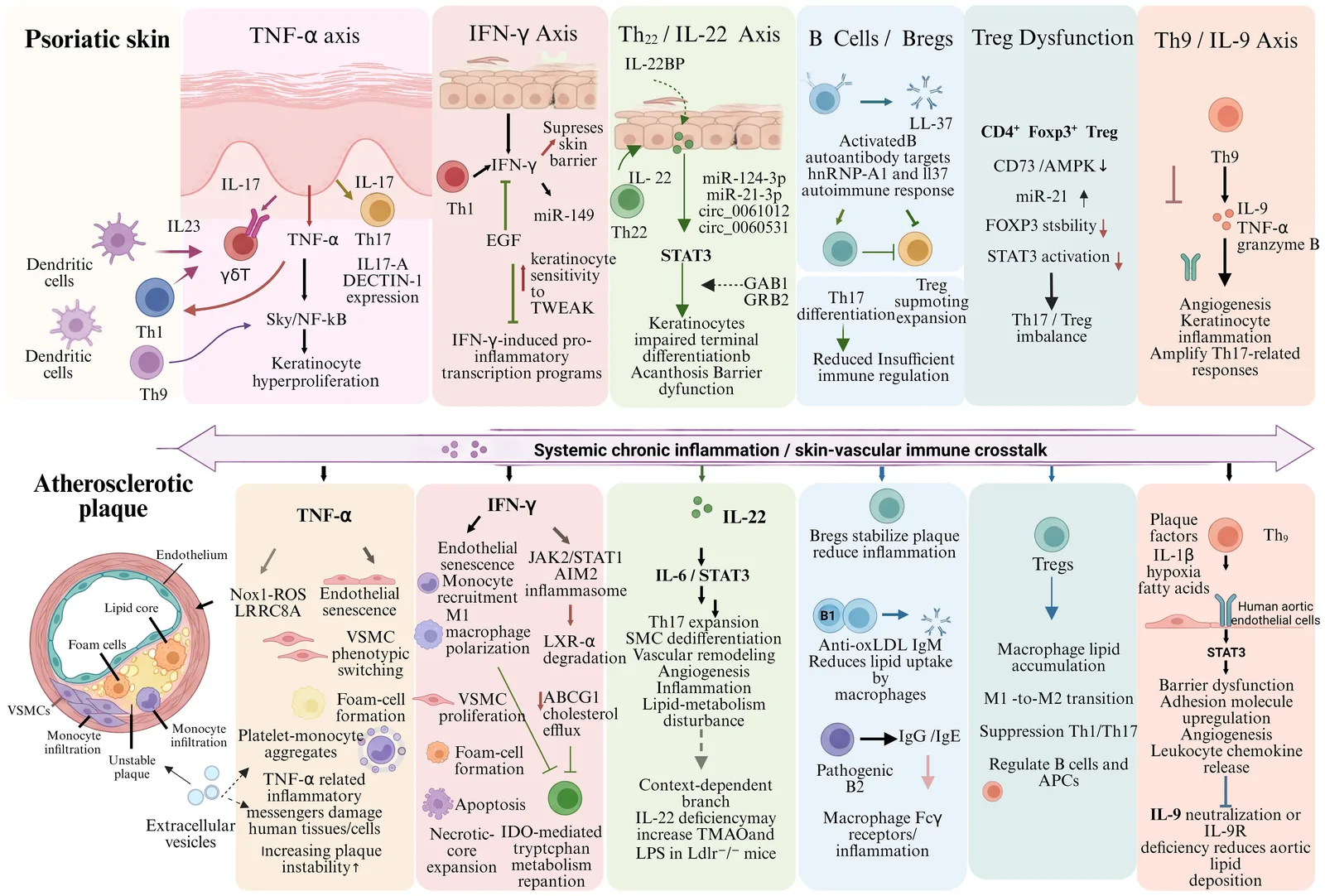
Skin-vascular crosstalk mediated by a multicytokine immune network in psoriatic skin inflammation and atherosclerotic plaque formation. In psoriatic skin, TNF-α, IFN-γ, IL-22, and the Th9/IL-9 axis cooperatively sustain chronic inflammation: the TNF-α/IL-17/IL-23 axis promotes DC and T cell activation and drives KC proliferation; IFN-γ regulates the inflammatory program of KCs; IL-22 promotes epidermal hyperplasia; and impaired Treg function together with insufficient Bregs weakens immune tolerance. After entering the circulation, these inflammatory mediators contribute to systemic inflammation and act on the vasculature. Within atherosclerotic plaques, TNF-α induces endothelial senescence, platelet aggregation, and foam cell formation; IFN-γ activates the AIM2 inflammasome via JAK2/STAT1 signaling, thereby promoting macrophage M1 polarization and expansion of the necrotic core; IL-22 influences vascular smooth muscle cell phenotype through IL-6/STAT3 signaling; and the Th9/IL-9 axis damages endothelial barrier integrity through STAT3, promotes adhesion molecule expression and leukocyte recruitment, and accelerates plaque destabilization. By contrast, Bregs and Tregs generally exert protective effects, and their functional loss may exacerbate these pathogenic processes. TNF-α, tumor necrosis factor-alpha; IFN-γ, interferon-gamma; IL-22, interleukin-22; Th9, T helper 9; IL-9, interleukin-9; IL-17, interleukin-17; IL-23, interleukin-23; Treg, regulatory T cells; Bregs, regulatory B cells; JAK2, Janus kinase 2; STAT1, signal transducer and activator of transcription 1; AIM2, absent in melanoma 2; M1, classically activated macrophages; IL-6, interleukin-6; STAT3, signal transducer and activator of transcription 3. Created with BioRender.com.

In AS, Th9 cell differentiation is regulated by the plaque microenvironment, including IL-1β, hypoxia, and fatty acids (111), whereas immune cells within human plaques express the interleukin-9 receptor (IL-9R), allowing local amplification of IL-9 signaling (112). Notably, recent studies have shown that Th9 cells exhibit vascular homing properties and can be detected in coronary artery plaques from patients with PSO. In a psoriasis-associated AS mouse model, neutralization of IL-9 or deficiency of IL-9R reduces aortic lipid deposition. Mechanistically, IL-9 can directly act on human aortic endothelial cells through the IL-9R/STAT3 signaling pathway, thereby inducing barrier dysfunction, upregulation of adhesion molecules, enhanced angiogenesis, and release of leukocyte chemokines (61). These findings move the Th9/IL-9 axis beyond purely correlative observations toward an evidence chain supported by clinical association, animal intervention, and cellular mechanistic data. In addition, in psoriatic arthritis (PsA), glucocorticoid-induced tumor necrosis factor receptor-related protein (GITR)/GITR ligand (GITRL) costimulatory signaling can promote the expansion of Th9 and Th17 cells, while GITR-positive helper T cells highly express the gut-homing molecule alpha 4 beta 7 (α4β7), suggesting that Th9 cells may participate in immune crosstalk among the skin, gut, and joints (113). Overall, compared with classical T cell subsets such as Th1, Th17, or Treg, the evidence base for the Th9/IL-9 axis in PSO and AS remains relatively limited. Current studies more strongly support its potential role as a cooperative amplifier within the inflammatory network rather than as a fully established core pathogenic driver. Future studies should clarify the cellular origin of Th9 cells, the target cells of IL-9, the disease stage-specific effects of this axis, and whether it forms a causal synergistic relationship with the IL-23/Th17 axis and the gut. The immune mechanisms discussed in Sections 3.2-3.5 are collectively summarized in **Figure 3**.

## 4 Clinical and therapeutic implications

Substantial epidemiological and clinical evidence demonstrates (**Table 2**) a strong positive association between the severity and duration of PSO and the risk of CVD. This highlights that patients with moderate-to-severe PSO constitute a high-risk population for CVD beyond cutaneous disease. Biologic therapies offer a unique form of “natural experiment” to evaluate the effect of immune regulation on cardiovascular outcomes.

**Table 2:** Effects of drugs targeting shared therapeutic targets on psoriasis and cardiovascular risk.

Therapeutic Target	Representative Drugs	Efficacy in psoriasis	Evidence for vascular inflammation and CVD risks	References
IL-17A/IL-17R	Secukinumab, Ixekizumab	Highly efficacious with rapid skin lesion clearance	Dual role: reduces non-calcified plaque burden yet may exert paradoxical effects on plaque stability, with an overall neutral impact on MACE risk	(114–117)
JAK inhibitors	Ruxolitinib, Tofacitinib, TG101348	Suitable for patients with prominent articular symptoms, and its efficacy against moderate-to-severe skin lesions is potentially inferior to that of IL-17/IL-23 inhibitors	Basic studies demonstrate anti-atherosclerotic potential, while its clinical cardiometabolic effects are complex: it elevates conventional blood lipids yet improves lipoprotein subtypes, and may increase the risk of MACE and venous thrombosis in high-risk populations	(118–120)
PCSK9 inhibitors	Alirocumab, Evolocumab	May reduce circulating IL-17 levels, alleviate cutaneous inflammation, and prevent or ameliorate psoriasis	Potently reduces LDL-C, attenuates atherosclerotic plaques and lowers cardiovascular event risks.	(121)
Statins	Atorvastatin, Simvastatin	Exerts anti-inflammatory effects and improves skin lesions (PASI/DLQI) in some patients	Lipid-lowering, anti-inflammatory, plaque-stabilizing, and reduces ASCVD risk	(122, 123)

CVD, cardiovascular disease; IL, interleukin; IL-17R, interleukin-17 receptor; JAK, Janus kinase; PASI, Psoriasis Area and Severity Index.

### 4.1 Impact of cardiovascular medications on psoriasis

#### 4.1.1 PCSK9 inhibitors

Proprotein convertase subtilisin/kexin type 9 (PCSK9) facilitates degradation of the low-density lipoprotein receptors (LDLRs), thereby impairing lipid metabolism. Levels of circulating PCSK9 are dramatically increased in patients with PSO and might contribute to the increased cardiovascular risk. Hyperlipidemic mouse models show that PCSK9 deficiency reduces circulating IL-17 levels. *In vitro* studies have shown that recombinant PCSK9 induces macrophage activation and promotes TNF-α and IL-6 expression, whereas PCSK9 inhibition attenuates macrophage inflammatory responses via the NF-κB pathway. In an imiquimod-induced murine PSO model, both genetic deletion of PCSK9 and siRNA-mediated knockdown alleviate disease severity. Current evidence suggests that approved PCSK9 inhibitors may have potential preventive effects in PSO (121). Moreover, beyond cholesterol lowering, PCSK9 inhibition may exert anti-inflammatory effects by decreasing systemic IL-17 levels, although its preventive role in PSO requires further clinical confirmation (121).

#### 4.1.2 Statins

Statins reduce LDL-C levels by blocking cholesterol synthesis and increasing LDL-C receptor expression; therefore, they reduce atherosclerotic cardiovascular disease (ASCVD) risk.

Beyond cholesterol reduction, statins exert anti-inflammatory and immunomodulatory effects through mevalonate pathway-dependent and -independent mechanisms, including suppression of lymphocyte function-associated antigen-1 (LFA-1), TNF-α, IL-6, and VEGF, and have therefore drawn interest in PSO care (122). Some patients may have reductions in PSO Area and Severity Index (PASI) scores and improvements in Dermatology Life Quality Index (DLQI) with statins, according to clinical studies (123). However, meta-analyses have reported considerable variation in their effects on PSO severity and inflammatory markers (high-sensitivity C-reactive protein (hs-CRP), IL-6, and TNF-α), with potential differences between topical and systemic use (124). Further large-scale randomized controlled studies are needed to define their therapeutic relevance and the extent to which anti-inflammatory benefits are independent of cholesterol reduction.

### 4.2 Impact of antipsoriatic agents on cardiovascular diseases

#### 4.2.1 Methotrexate

Methotrexate (MTX) is a conventional systemic treatment for PSO, yet its effectiveness remains debatable. Furthermore, long-term observational studies suggest the continued efficacy and safety of low-dose MTX (125), but its efficacy is limited in refractory subtypes such as palmoplantar psoriasis. In combination therapy, MTX has shown efficacy when combined with apremilast (126), but synergistic benefits with biologics such as adalimumab have been inconsistent (127). This indicates that the efficacy of MTX is highly context dependent; therefore, clinical decision-making should involve an individualized balance among patient subtype, disease course, comorbidities, and therapeutic goals. In the cardiovascular setting, MTX can downregulate proinflammatory mediators such as TNF-α, interleukin-1 (IL-1), and IL-6, upregulate anti-inflammatory cytokines such as IL-10, and scavenge free radicals while enhancing antioxidant defenses, thereby potentially exerting a protective effect against AS (128). The Cardiovascular Inflammation Reduction Trial (CIRT), however, did not demonstrate cardiovascular benefit, perhaps because of folate supplementation. Future studies should separately evaluate MTX, folate, and combination therapy using suitable controls to understand their cardiovascular effects (129).

#### 4.2.2 Biologics

At present, ten biologic agents targeting IL-17, IL-23, TNF-α, and IL-12/23 pathways are available for PSO treatment. Compared with oral therapies, biologic treatment is generally associated with lower CVD risk (130).

TNF-α inhibitors: Adalimumab lowers levels of IL-6 and other inflammatory mediators associated with AS in PSO. However, the cardiovascular safety of adalimumab is still debatable (131). Adalimumab has been associated with thrombotic cardiovascular risk, whereas other TNF inhibitors such as golimumab and etanercept did not exhibit similar findings (132).

IL-23 inhibitors: IL-23 inhibitors have shown excellent drug survival in the treatment of PSO, especially drugs such as risankizumab (61). However, data on cardiovascular risk reduction remain inadequate and contradictory. Animal studies have not definitively demonstrated an anti-atherosclerotic effect of IL-23 inhibition (130). A small nonrandomized observational trial indicated a possible improvement in arterial stiffness with IL-23 suppression, but the data need to be viewed with caution (133). Thus, the cardiovascular effects of IL-23 inhibitors are not yet clearly defined.

IL-17 inhibitors: Despite their excellent efficacy, IL-17 inhibitors for PSO treatment still face challenges from complex cutaneous adverse reactions and unknown cardiovascular consequences (114). Two main types of cutaneous adverse effects have been described. The first involves changes in the PSO phenotype or newly developed eczema/atopic dermatitis, which could be associated with reduced Th1/Th17 cytokine signaling and an increase in Th2 immunity following IL-17 inhibition (134). The second is classical paradoxical PSO, probably mediated by inhibition of TNF-α, activation of pDCs, and unregulated production of IFN-α (135). These events are usually addressed more aggressively clinically than TNF inhibitor-associated reactions, and re-challenge with IL-17 inhibitors is not indicated. The potential therapeutic role of JAK inhibitors in such conditions remains to be elucidated (135).

IL-17 has paradoxical effects in AS, presumably driving inflammatory progression but also contributing to plaque stability (115). This dual role is supported by both animal and clinical research. IL-17 inhibitors have been demonstrated to lower the burden of non-calcified plaque in patients with PSO, suggesting a pro-atherogenic effect of IL-17 (116). By contrast, certain clinical studies correlate low IL-17 levels with unfavorable cardiovascular outcomes (117). These inconsistencies may reflect differences in disease stage, experimental models, and study design. Although IL-17/interleukin-17 receptor (IL-17R) inhibitors have not been clearly associated with increased major cardiovascular events, their long-term effects on endothelial function and plaque progression remain uncertain, and systematic evidence synthesis is lacking. Further studies are needed to clarify the mechanistic and therapeutic role of IL-17 in AS.

JAK inhibitors: JAK inhibitors have emerged as a mainstay therapeutic approach in immune-mediated disorders with agents such as upadacitinib and tofacitinib approved for PSO. Phase III trials suggest they may be less effective for moderate-to-severe skin disease than IL-17/IL-23 inhibitors, but may offer particular benefit in those with considerable joint involvement (118). Preclinical investigations show potential antiatherosclerotic benefits. The JAK2 inhibitor TG101348 attenuated the progression of AS (119), whereas ruxolitinib reduced plaque burden and tofacitinib inhibited monocyte adhesion and promoted cholesterol efflux (120). However, JAK inhibitors may increase conventional cholesterol levels in a dose-dependent manner, despite potentially favorable alterations in lipoprotein subfractions such as LDL particle density (136). Clinical evidence, notably in rheumatoid arthritis patients, suggests that tofacitinib is associated with increased risk for major adverse cardiovascular events (MACE) and venous thromboembolism (137). Therefore, the decision to treat should balance anti-inflammatory and prospective vascular advantages against cardiovascular risk, particularly in patients at high risk.

### 4.3 Effects of drugs commonly associated with both conditions

#### 4.3.1 Vitamin D

Topical vitamin D analogs are a conventional and effective treatment for PSO, while the therapeutic benefit of oral vitamin D supplementation remains uncertain (138). Although PSO patients generally exhibit lower serum 25-hydroxyvitamin D (25(OH)D) levels than healthy individuals, and some observational studies suggest an inverse correlation with PASI scores, oral vitamin D supplementation has not consistently resulted in significant PASI improvement (139). These discrepancies may reflect population heterogeneity, limited sample size, interindividual variation in vitamin D metabolism, and potential confounding factors.

Vitamin D deficiency in AS may promote disease progression through several routes, including greater expression of adhesion molecules, accelerated recruitment of immune cells, oxidative stress, vascular tone alteration, and suppression of VSMC proliferation (140). Vitamin D also modulates macrophage cholesterol metabolism and polarization, thereby affecting plaque formation and stability (141, 142). Vitamin D deficiency might be a potential link between PSO and CVD, but there is still a lack of evidence to support vitamin D as a reliable marker of cardiovascular risk in PSO.

## 5 Discussion

**Figure 4:**
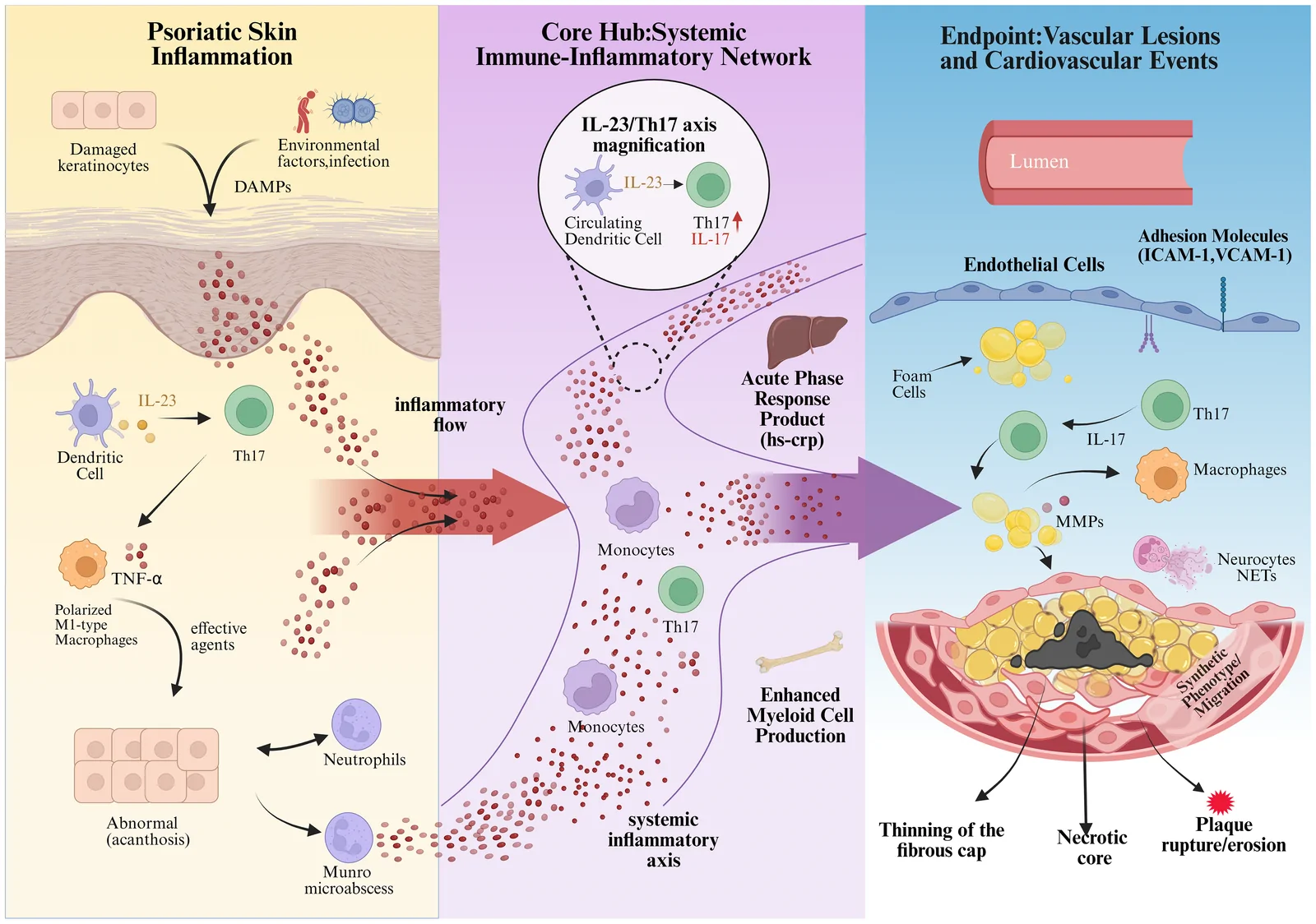
Integrated model — the “immune continuum” from skin inflammation to cardiovascular event. KCs release DAMPs that activate DCs and induce IL-23 production, promoting Th17 differentiation and IL-17 secretion. These mediators enter the circulation and amplify systemic inflammation and acute-phase responses. Monocytes and Th17 cells induce endothelial activation by upregulating ICAM-1 and VCAM-1. Monocytes subsequently differentiate into macrophages and form foam cells, promoting plaque formation. NETs and IL-17 further exacerbate inflammation, leading to fibrous cap thinning, necrotic core formation, and plaque instability. DAMPs, damage-associated molecular patterns; IL, interleukin; Th17, Thelper 17 cells; ICAM-1, intercellular adhesion molecule-1; VCAM-1, vascular cell adhesion molecule-1; NETs, neutrophil extracellular traps. Created with BioRender.com.

This review systematically summarizes the shared mechanisms of innate and adaptive immunity in PSO and AS (**Figure 4**), suggesting that these two conditions are not merely simple comorbidities but may represent a systemic immune continuum driven by highly overlapping immunoinflammatory networks. At the level of innate immunity, DCs initiate inflammatory responses through danger signal recognition, IFN-I release, and IL-23-mediated T cell polarization; neutrophils release damage-associated signals through ROS, NETs, and pyroptosis; macrophages exhibit M1/M2 and multi-subtype plasticity and participate in foam cell formation and plaque destabilization; and inflammasomes, mast cells, and ILCs further integrate injury-related, neural, immune, and metabolic signals, together forming an innate immune bridge that links cutaneous inflammation with vascular injury.

At the level of adaptive immunity, the IL-23/Th17 axis occupies a central position in linking PSO with AS. Activated DCs drive Th17 expansion through IL-23 and promote the release of IL-17A, which cooperates with TNF-α, IFN-γ, and IL-22 to act on KCs, vascular endothelial cells, VSMCs, and macrophages, thereby establishing an inflammatory amplification loop spanning the skin and vasculature. Meanwhile, imbalance among Th1, Th17, and Treg, impaired Breg function, and activation of pathogenic B cells further weaken immune tolerance and sustain chronic inflammation. Notably, cutaneous inflammation may also reshape specific T cell subsets, particularly the Th9/IL-9 axis, enabling these cells to acquire vascular homing and endothelial injury-related properties. Through IL-9R/STAT3 signaling, this axis can induce endothelial barrier disruption, upregulation of adhesion molecules, and leukocyte chemotaxis, thereby serving as a cooperative amplification pathway for PSO-associated spillover into vascular inflammation. However, IL-17 and IL-23 should not be regarded as fixed, unidirectional proinflammatory mediators. Although the IL-23/IL-17 axis has a well-established pathogenic role in PSO, its effects in AS are highly context dependent. On the one hand, this axis can promote endothelial dysfunction, inflammatory cell recruitment, foam cell formation, and intraplaque inflammatory amplification; on the other hand, under specific tissue locations, disease stages, or homeostatic conditions, IL-23/IL-17 signaling may also contribute to intestinal barrier maintenance, inflammatory containment, or structural stabilization of plaques (49, 70, 143). Similarly, IL-22 can both sustain Th17-associated inflammatory responses and regulate VSMC dedifferentiation and plaque remodeling (53, 144).

Therefore, we propose that psoriatic skin inflammation may systemically preactivate myeloid and T cell programs at an early stage; however, the ultimate vascular consequences of these immune signals depend on plaque stage, local metabolic niche, host background, and the type of secondary stimulation. In early or active plaques, IL-23/Th17-related signaling is more likely to promote endothelial activation, adhesion molecule expression, leukocyte recruitment, oxidative stress, and foam cell formation, thereby accelerating plaque initiation and inflammatory amplification. By contrast, in advanced or fibrotic plaques, the same pathway may also influence vascular smooth muscle cell phenotype, collagen deposition, matrix remodeling, fibrous cap integrity, and necrotic core evolution. Accordingly, in some apolipoprotein E-deficient (*ApoE-/-*) models, IL-17 exerts a proatherogenic effect, whereas in certain clinical settings, low IL-17 activity is associated with adverse cardiovascular outcomes. This likely reflects a functional shift of the same immune program across different temporal windows and tissue niches, rather than a true biological contradiction.

Animal models and dietary context are not neutral technical variables, but important determinants of immune phenotypes. Current PSO-associated AS models include the Rac1V12 model, the triple-genetically engineered model (*Srb1-/-/ApoeR61H/H/K14-Rac1V12-/+*), the imiquimod (IMQ)-induced model, the IL-23-induced model, the Card14-related model, and translational models based on monogenic human PSO (145, 146). These models differ substantially in genetic background, lipid metabolism, mode of inflammatory triggering, disease duration, and plaque severity. Moreover, most of them mimic acute cutaneous inflammation or monogenic disease-driven lesions and therefore cannot fully recapitulate the pathological context of human PSO, which is characterized by long-term, low-grade, recurrent inflammation and the superimposition of multiple CVD risk factors.

Meanwhile, *Apoe-/-* and (*Ldlr-/-*) backgrounds generate distinct lipid profiles, plaque compositions, and inflammatory set points; likewise, differences in Western diet, high-fat diet, or high-cholesterol diet regimens, as well as feeding duration, can reshape the gut microbiota, circulating lipids, TMAO, LPS exposure, and systemic inflammatory status. Therefore, inconsistencies among experimental findings may more likely reflect model-specific immunometabolic states rather than mutual negation of a single pathway’s effects.

Population heterogeneity in clinical studies may likewise weaken causal inference. Age, sex, ethnicity, body mass index (BMI), smoking status, diabetes mellitus, hypertension, dyslipidemia, PSO duration and severity, concomitant PsA, prior exposure to biologics, statin use, and other immunomodulatory therapies can all modify systemic inflammatory burden and CVD outcomes. Because the current clinical evidence is largely derived from observational studies, the inflammation-mediated effects of PSO remain difficult to fully distinguish from traditional cardiovascular risk factors. Therefore, the predictive value of candidate biomarkers such as TNF-α, IL-6, and IL-17, the degree of assay standardization, and their incremental value over existing CVD risk scores still require validation in large prospective cohorts and stratified studies (147).

## 6 Conclusion

In conclusion, molecular and clinical evidence support the hypothesis that PSO and AS are not coincidental comorbidities, but rather a continuum of pathological processes driven by a systemic immunoinflammatory network centered on the IL-23/Th17 axis. The “immune continuum” paradigm highlights the underlying connection between skin and vascular inflammation and redefines PSO as a systemic inflammatory disease with cardiovascular spillover consequences beyond skin involvement.

Biologic agents targeting TNF-α, IL-17, and IL-23 may confer cardiovascular benefits beyond their effects on cutaneous inflammation, potentially through reducing systemic inflammatory burden and improving endothelial function. However, these effects are heterogeneous, with differential cardiovascular safety profiles across therapeutic targets and agents, as well as potential unfavorable signals in high-risk patients. These findings suggest that treatment decisions should go beyond skin-directed control and incorporate immune-inflammatory phenotypes and cardiovascular risk stratification.

Current translational research is still transitioning from mechanistic association to causal validation. Immune-inflammatory biomarkers show promise, but evidence regarding their standardization, reproducibility, and incremental predictive value remains limited. Future studies should explore causal pathways within the “skin-vascular axis,” mechanisms of humoral signaling and inflammatory cell trafficking, disease-modifying effects of IL-23/Th17-targeted therapies on plaque progression in prospective imaging and clinical endpoint studies, and the role of trained immunity and hematopoietic reprogramming in long-term cardiovascular risk. PSO is essentially a systemic immune-inflammatory disease, and its therapeutic strategies are shifting from cutaneous control toward integrated intervention targeting systemic inflammation and cardiovascular risk. Immune phenotyping combined with conventional cardiovascular risk assessment may facilitate the transition from comorbidity management to mechanism-based precision medicine.
